# Portfolio of prospective clinical trials including brachytherapy: an analysis of the ClinicalTrials.gov database

**DOI:** 10.1186/s13014-016-0624-8

**Published:** 2016-03-22

**Authors:** Nikola Cihoric, Alexandros Tsikkinis, Cristina Gutierrez Miguelez, Vratislav Strnad, Ivan Soldatovic, Pirus Ghadjar, Branislav Jeremic, Alan Dal Pra, Daniel M. Aebersold, Kristina Lössl

**Affiliations:** Department of Radiation Oncology, Inselspital, Bern University Hospital and University of Bern, 3010 Bern, Switzerland; Department of Radiation Oncology, Charité Universitätsmedizin Berlin, Berlin, Germany; Department of BT, ICO Hospitalet, Hospital Duran iReynals, l’Hospitalet de Llobregat, Barcelona, Spain; Institute of Lung Diseases, Sremska Kamenica, Serbia; Centre for Biomedical Research, BioIRC, Kragujevac, Serbia; Faculty of Medicine, University of Belgrade, Belgrade, Serbia; Department of Radiation Oncology, University Hospital Erlangen, Erlangen, Germany

**Keywords:** Brachytherapy, Clinicaltrials.gov, Interventional clinical trials

## Abstract

**Background:**

To evaluate the current status of prospective interventional clinical trials that includes brachytherapy (BT) procedures.

**Methods:**

The records of 175,538 (100 %) clinical trials registered at ClinicalTrials.gov were downloaded on September 2014 and a database was established. Trials using BT as an intervention were identified for further analyses. The selected trials were manually categorized according to indication(s), BT source, applied dose rate, primary sponsor type, location, protocol initiator and funding source. We analyzed trials across 8 available trial protocol elements registered within the database.

**Results:**

In total 245 clinical trials were identified, 147 with BT as primary investigated treatment modality and 98 that included BT as an optional treatment component or as part of the standard treatment. Academic centers were the most frequent protocol initiators in trials where BT was the primary investigational treatment modality (*p* < 0.01). High dose rate (HDR) BT was the most frequently investigated type of BT dose rate (46.3 %) followed by low dose rate (LDR) (42.0 %). Prostate was the most frequently investigated tumor entity in trials with BT as the primary treatment modality (40.1 %) followed by breast cancer (17.0 %). BT was rarely the primary investigated treatment modality for cervical cancer (6.8 %).

**Conclusion:**

Most clinical trials using BT are predominantly in early phases, investigator-initiated and with low accrual numbers. Current investigational activities that include BT mainly focus on prostate and breast cancers. Important questions concerning the optimal usage of BT will not be answered in the near future.

**Electronic supplementary material:**

The online version of this article (doi:10.1186/s13014-016-0624-8) contains supplementary material, which is available to authorized users.

## Introduction

Radiotherapy (RT) was first applied in medicine at the beginning of the 20th century [1]. Nowadays RT plays an ever increasing role in the treatment of multiple tumor entities, with more than 50 % of all cancer patients receiving some sort of RT, in curative or palliative intent, during the course of their disease [2]. Brachytherapy (BT) has been a well-established modality, mainly due to its high conformity and possibility of sparing organs-at-risk [3]. The field of RT has recently seen the introduction of several new technologies in everyday praxis. However, the utilization of newer technologies is not always accompanied with a high level of evidence [4]. Furthermore, little is known about ongoing prospective research in RT. This holds true for BT as well.

Several clinical trial registries were established during the past decade. Trial registration is being regulated with European and US federal laws as well as international conventions (World Health Organization, WHO) [5, 6]. Registration of all interventional clinical trials is obligatory in the European Union (EU) and the United States (US) and is demanded by an international consortium of medical journal editors [7, 8]. ClinicalTrials.gov is the largest clinical trial registry with over 190,000 registered trials and a high weekly growth rate of new registrations. The registration process and its potential for an in-depth analysis of the clinical trials landscape is well described in the literature [6, 9–11]. Several studies evaluating different disease groups have already been published [12–14].

Goal of this study was to evaluate the present landscape of interventional clinical trials that include BT alone or as part of a multimodal approach.

## Material and methodes

### Data acquisition

**Fig. 1:**
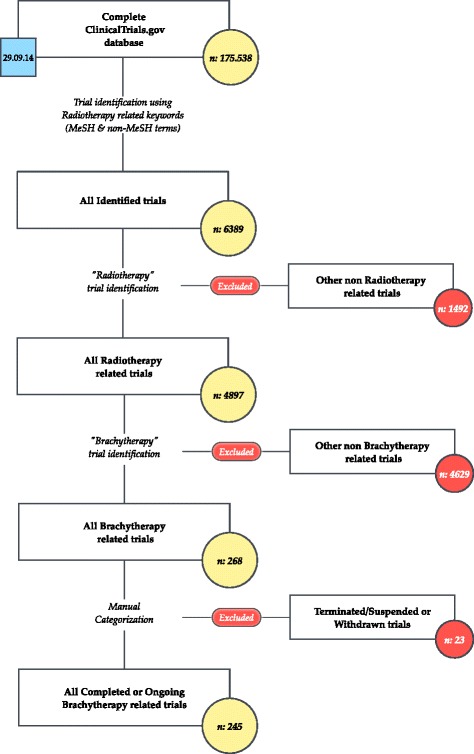
Trial selection process

The xml [15] records of 175,538 (100 %) clinical trials registered at ClinicalTrials.gov were downloaded on September 29th 2014 and a database was created. The established database was searched using MeSH terms and a list of keywords. In total, 6389 (3.6 %) trials were identified and selected for further manual review with 4897 (2.8 %) being radiotherapy related. We stratified trials according to the utilized radiotherapy technique. BT was part of the investigated procedure in 268 (0.1 %) trials. Suspended, terminated or withdrawn trials were excluded from further analysis (*n* = 23). In total, 245 trials were identified. The trial selection process is shown in Fig. 1.

We categorized the trials according to the role of BT procedures into two categories: 1. trials with BT in focus as primary investigated treatment modality (BF, *n* = 147); 2. trials in which external beam radiotherapy (EBRT) was combined with BT and trials, where the usage of BT was optional, the “other trials” Group (OT, *n* = 98).

Trials were manually categorized according to the investigated conditions based on the International Classification of Diseases (ICD-10).

We classified the primary sponsor and collaborators in the following categories: National Institute of Health - USA (NIH), Academic Institutions, Cooperative Groups and Industry. The primary sponsor field was used to categorize the protocol initiator.

Probable funding source (source of monetary support) was determined based on a modified methodology described in the work of Hirsch et al. and Califf et al. [16, 17] (Appendix).

Basic information of the selected trials, including the protocol initiator, funding source, BT dose rate type, countries where the trials were conducted and bodily organ investigated are presented in Table 1.

**Table 1:** Protocol initiator, source of founding, nature of BDR, organ and state

	All	BF	OT	*p* value
	(*n* = 245, 100 %)	(*n* = 147, 60 %)^c^	(*n* = 98, 40 %)^d^	
Protocol initiator				
Academic	176 (71.8 %)	120 (81.6 %)	56 (57.1 %)	<0.001
Collaborative Groups	42 (17.1 %)	11 (7.5 %)	31 (31.6 %)	
Industry	15 (6.1 %)	13 (8.8 %)	2 (2.0 %)	
NIH	12 (4.9 %)	3 (2.0 %)	9 (9.2 %)	
Source of funding				
Academic	145 (59.2 %)	99 (67.3 %)	46 (46.9 %)	<0.001
Collaborative Groups	13 (5.3 %)	2 (1.4 %)	11 (11.2 %)	
Industry	30 (12.2 %)	25 (17.0 %)	5 (5.1 %)	
NIH	56 (22.9 %)	20 (13.6 %)	36 (36.7 %)	
Public-private part.	1 (0.4 %)	1 (0.7 %)	0 (0.0 %)	
Type of BDR^a^				
Not defined^b^	83 (33.9 %)	30 (20.4 %)	53 (54.1 %)	<0.001
HDR	75 (46.3 %)	53 (45.3 %)	22 (48.9 %)	0.001
HDR/LDR	16 (9.9 %)	6 (5.1 %)	10 (22.2 %)	
HDR/PDR	2 (1.2 %)	2 (1.7 %)	0 (0.0 %)	
LDR	68 (42.0 %)	56 (47.9 %)	12 (26.7 %)	
PDR	1 (0.6 %)	0 (0.0 %)	1 (2.2 %)	
Country				
USA	144 (58.8 %)	85 (57.8 %)	59 (60.2 %)	0.219
Canada	27 (11.0 %)	20 (13.6 %)	7 (7.1 %)	
France	10 (4.1 %)	6 (4.1 %)	4 (4.1 %)	
China	10 (4.1 %)	7 (4.8 %)	3 (3.1 %)	
Germany	10 (4.1 %)	8 (5.4 %)	2 (2.0 %)	
Other	44 (18.0 %)	21 (14.3 %)	23 (23.5 %)	
Organ				
Prostate	80 (32.7 %)	59 (40.1 %)	21 (21.4 %)	<0.001
Cervix uteri	54 (22.0 %)	10 (6.8 %)	44 (44.9 %)	
Breast	29 (11.8 %)	25 (17.0 %)	4 (4.1 %)	
Uterus	14 (5.7 %)	5 (3.4 %)	9 (9.2 %)	
Other	68 (27.8 %)	48 (32.7 %)	20 (20.4 %)	

^a^ *BDR* brachytherapy dose rate, *HDR* high dose rate, *LDR* low dose rate, *PDR* pulse dose rate

^b^Percentage of total number per column

^c^Brachytherapy in focus

^d^Other trials

### Statistical analysis

Descriptive data are presented in numbers and percentages. Differences between the two groups were analyzed using the Pearson chi-square test and Chi-Square test. Statistical analysis was conducted using the SPSS software version 20.0 (SPSS Inc., Chicago, IL, USA). All p values less than 0.05 were considered significant.

### Results

Our study included 245 clinical trials, 147 trials with BT in focus (BF group, 60 %) and 98 other trials (OT group, 40 %).

Academy was the most frequent protocol initiator and source of funding. Focused on the protocol initiator, academy is dominant in the BF group but equal with NIH in the OT group. Collaborative groups have a higher percentage in other trials, compared to BT. The type of BT dose rate (BDR) differs significantly in the BF group as compared to the OT group. Missing data (not defined) is twofold higher in the OT group. Prostate is the most researched treated site in the BF group, whereas Cervix Uteri is the most studied site in the OT group. No significant differences were seen between all trials, BF and OT groups according to the countries where the trials were conducted (p: 0.355) (Table 1).

When comparing data availability, registered information, such as trial phase, is predominantly missing from the BF Group, whereas in all other investigated parameters the OT group has a higher data unavailability (Tables 2 and 3). The groups differ in trial phase, endpoint classification and allocation. Phase 2 trials are the most common in both the BF and OT groups (41.6 and 36.4 %, respectively) followed by phase 3 trials (23 and 33 %, respectively). The groups differ in the percentage of phase 1 and phase 4 trials, but have a similar distribution of trial arms, enrollment and intervention model (Table 2).

**Table 2:** Trial characteristic

	All	BF	OT	Availability^*^
	(*n* = 245, 100 %)	(*n* = 147, 60 %)^b^	(*n* = 98, 40 %)^c^	BF vs OT^**^
Trial phase				
Data not available^a^	44 (18.0 %)	34 (23.1 %)	10 (10.2 %)	0.010^*^
Phase 0	1 (0.5 %)	1 (0.9 %)	0 (0.0 %)	0.005^**^
Phase 1	33 (16.4 %)	13 (11.5 %)	20 (22.7 %)	
Phase 1/Phase 2	16 (8.0 %)	10 (8.8 %)	6 (6.8 %)	
Phase 2	79 (39.3 %)	47 (41.6 %)	32 (36.4 %)	
Phase 2/Phase 3	4 (2.0 %)	4 (3.5 %)	0 (0.0 %)	
Phase 3	55 (27.4 %)	26 (23.0 %	29 (33.0 %)	
Phase 4	13 (6.5 %)	12 (10.6 %)	1 (1.1 %)	
Number of trial arms				
Data not available^a^	48 (19.6 %)	28 (19.0 %)	20 (20.4 %)	0.793^*^
1	110 (55.8 %)	71 (59.7 %)	39 (50.0 %)	0.081^**^
2	74 (37.6 %)	43 (36.1 %)	31 (39.7 %)	
3	6 (3.0 %)	2 (1.7 %)	4 (5.1 %)	
4	6 (3.0 %)	3 (2.5 %)	3 (3.8 %)	
5	1 (0.5 %)	0 (0.0 %)	1 (1.3 %)	
Enrollment (No of patients)				
Data not available^a^	2 (0.8 %)	9 (6.1 %)	7 (7.1 %)	0.751^*^
1–50	105 (43.2 %)	65 (44.8 %)	40 (40.8 %)	0.184^**^
51–100	40 (16.5 %)	26 (17.9 %)	14 (14.3 %)	
101–200	27 (11.1 %)	15 (10.3 %)	12 (12.2 %)	
201–500	30 (12.3 %)	20 (13.8 %)	10 (10.2 %)	
>500	41 (16.9 %)	19 (13.1 %)	22 (22.4 %)	
Intervention model				
Data not available^a^	40 (16.3 %)	18 (12.2 %)	22 (22.4 %)	0.034^*^
Single Group Assignment	124 (60.5 %)	84 (65.1 %)	40 (52.6 %)	0.075^**^
Parallel Assignment	78 (38.0 %)	42 (32.6 %)	36 (47.4 %)	
Factorial Assignment	2 (1.0 %)	2 (1.6 %)	0 (0 %)	
Crossover Assignment	1 (0.5 %)	1 (0.8 %)	0 (0 %)	
Endpoint classification				
Data not available^a^	74 (30.2 %)	38 (25.9 %)	36 (36.7 %)	0.069^*^
Safety Study	18 (10.5 %)	5 (4.6 %)	13 (21.0 %)	0.002^**^
Efficacy Study	60 (35.1 %)	42 (38.5 %)	18 (29.0 %)	
Safety/Efficacy Study	91 (53.2 %)	61 (56.0 %)	30 (48.4 %)	
Bio-equivalence Study	1 (0.6 %)	1 (0.9 %)	0 (0.0 %)	
Pharmacodynamics Study	1 (0.6 %)	0 (0 %)	1 (1.6 %)	

^a^Percents of total number per column

^b^Brachytherapy in focus

^c^Other trials

^*^ *p* value for availability of data between groups

^**^ *p* value for test between BF and OT (without unavilable data)

**Table 3:** Trial characteristics-continued

	All	BF	OT	Availability^*^
	(*n* = 245, 100 %)	(*n* = 147, 60 %)^b^	(*n* = 98, 40 %)^c^	BF vs OT^**^
Primary purpose				
Data not available^a^	7 (2.9 %)	4 (2.7 %)	3 (3.1 %)	1.000^*^
Treatment	214 (89.9 %)	126 (88.1 %)	88 (92.6 %)	0.550^**^
Supportive Care	8 (3.4 %)	6 (4.2 %)	2 (2.1 %)	
Diagnostic	9 (3.8 %)	6 (4.2 %)	3 (3.2 %)	
Health Services Res.	3 (1.3 %)	3 (2.1 %)	0 (0 %)	
Prevention	4 (1.7 %)	2 (1.4 %)	2 (2.1 %	
Masking				
Data not available^a^	37 (15.1 %)	19 (12.9 %)	18 (18.4 %)	0.244^*^
Open Label	195 (93.8 %)	117 (91.4 %)	78 (97.5 %)	0.105^**^
Double-Blind	6 (2.9 %)	4 (3.1 %)	2 (2.5 %)	
Single Blind	7 (3.4 %)	7 (5.5 %)	0 (0 %)	
Allocation				
Data not available^a^	111 (45.3 %)	65 (44.2 %)	46 (46.9 %)	0.675^*^
Non-Randomized	44 (32.8 %)	31 (37.8 %)	13 (25.0 %)	0.124^**^
Randomized	90 (67.2 %)	51 (62.2 %)	39 (75.0 %)	
Overall				
Active, not recruiting	52 (21.2 %)	34 (23.1 %)	18 (18.4 %)	0.075^**^
Completed	83 (33.9 %)	40 (27.2 %)	43 (43.9 %)	
Recruiting	96 (39.2 %)	62 (42.2 %)	34 (34.7 %)	
Not yet recruiting	13 (5.3 %)	10 (6.8 %)	3 (3.1 %)	
Enrolling by invitation	1 (0.4 %)	1 (0.7 %)	0 (0 %)	

^a^Percentage of total number per column

^b^Brachytherapy in focus

^c^Other trials

^*^ *p* value for availability of data between groups

^**^ *p* value for test between BF and OT (without unavilable data)

Based on our results, shown in Table 3, the examined groups significantly differ in trial allocation. Randomized trials are more frequent in the OT group, whereas the BF group has mostly non-randomized trials. In the BF group, trials are mostly in a recruiting stage, while in the OT group they are mostly completed.

Focusing on the BF group alone (Table 4), there is a significant difference between the investigated organs, only during the last changed period. Although the median duration to the primary outcome and time to completion differs between organs, this difference is not statistically significant likely due to the high variability and small sample sizes.

**Table 4:** Organ in BF group

BF group	Prostate (*n* = 80, %)	Cervix uteri (*n* = 54, %)	Breast (*n* = 29, %)	Uterus (*n* = 14, %)	Others (*n* = 68, %)	*p* value
Prim. Completion attribute						
Actual	30 (37.5 %)	18 (33.3 %)	13 (44.8 %)	3 (21.4 %)	18 (26.5 %)	0.283^a^
Anticipated	43 (53.8 %)	29 (53.7 %)	15 (51.7 %)	9 (64.3 %)	36 (52.9 %	
None	7 (8.8 %)	7 (13.0 %)	1 (3.4 %)	2 (14.3 %)	14 (20.6 %)	
Completion date attribute						
Actual	26 (32.5 %)	12 (22.2 %)	8 (27.6 %)	3 (21.4 %)	20 (29.4 %)	0.112^a^
Anticipated	39 (48.8 %)	19 (35.2 %)	15 (51.7 %)	5 (35.7 %)	24 (35.3 %)	
None	15 (18.8 %)	23 (42.6 %)	6 (20.7 %)	6 (42.9 %)	24 (35.3 %)	
First received						
1999–2004	12 (15.0 %)	11 (20.4 %)	1 (3.4 %)	1 (7.1 %)	19 (27.9 %)	<0.001^a^
2005–2009	29 (36.2 %)	19 (35.2 %)	19 (65.5 %)	10 (71.4 %)	20 (29.4 %)	
2010–2015	39 (48.8 %)	24 (44.4 %)	9 (31.0 %)	3 (21.4 %)	29 (42.6 %)	
Last changed						
2005–2009	4 (5.0 %)	4 (7.4 %)	2 (6.9 %)	0	12 (17.6 %)	0.202^a^
2010–2015	76 (95.0 %)	50 (92.6 %)	27 (93.1 %)	14 (100 %)	56 (82.4 %)	
Primary completion						
2000–2004	4 (5.5 %)	0	1 (3.6 %)	0	7 (13.0 %)	0.104^a^
2005–2009	10 (13.7 %)	10 (21.3 %)	6 (21.4 %)	0	6 (11.1 %)	
2010–2015	36 (49.3 %)	27 (57.4 %)	17 (60.7 %)	8 (66.7 %)	28 (51.9 %)	
2016+	23 (31.5 %)	10 (21.3 %)	4 (14.3 %)	4 (33.3 %)	13 (24.1 %)	
Completion year						
2000–2004	2 (3.0 %)	0	0	0	6 (13.0 %)	0.027^a^
2005–2009	9 (13.6 %)	9 (27.3 %)	2 (8.3 %)	1 (12.5 %)	9 (19.6 %)	
2010–2015	31 (47.0 %)	16 (48.5 %)	11 (45.8 %)	5 (62.5 %)	17 (37.0 %)	
2016+	24 (36.4 %)	8 (24.2 %)	11 (45.8 %)	2 (25.0 %)	14 (30.4 %)	

^a^Chi square test

## Discussion

We have shown that BT is rarely evaluated in prospective clinical trials either as a single treatment modality or combined with EBRT. There are several possible explanations for this occurrence. BT is a demanding discipline, because of the need for experienced personnel and the specialized equipment required. Even in centers with available infrastructure, BT is most often used for the treatment of gynecological malignancies and rarely used for other tumor entities [18–21]. Furthermore, new highly conformal EBRT techniques are gaining in popularity, although their usage is still not based on high quality evidence [22–24]. This, in turn, potentially affected and caused the decline of BT as a viable treatment modality. Unfortunately, the current trend continues and is even seen in tumor entities where BT is considered mandatory [25, 26]. All of the above makes patient accrual difficult.

Differences between the BF and OT groups are seen in protocol development and trial funding. 81.6 % of initiated protocols in BF group and 57.1 % in the OT group have academic institutions as a primary funding source and protocol initiators. There appears to be limited interest from the National Institute of Health – USA (NIH), the industry and collaborative groups. This effectively shifts the monetary burden to academic institutions, with little support from the industry or NIH. Compared to the general oncological landscape of trial funding [17], we see that 41.8 % of trials were primarily industry-funded, 15.3 % government-funded (NIH) and 42.9 % from predominantly academic sources.

Most trials were initiated in the US (58.8 %), followed by Canada (11.0 %) and France (4.1 %). A possible explanation is that registration of trials in the ClinicalTrials.gov registry is obligatory in the US. Nevertheless, the ClinicalTrials.gov registry encompasses more than 70 % of all trials registered in the primary WHO registry. Also the uneven distribution could be a result of the lower expenditure for healthcare research in the EU [27, 28].

A major problem of this study is the low data availability. Data availability appears higher in trials initiated and sponsored by academic institutions and in BT focused trials. Individual investigators or institutions seem more eager to submit data during the registration process. However, in the work from Califf et al. it is shown that data availability shows a positive time trend, with more data becoming available as registration progresses [16]. In addition some data are more accurate within the database as compared to the published data [29, 30]. Sponsors should be motivated to submit accurate and updated data into the registries.

### Further discussion-diseases

#### Cervical cancer (CCa)

Although CCa is the tumor entity most commonly treated with combined EBRT and BT in clinical practice, prospective trials investigating the role of BT in the treatment of CCa are surprisingly rare, especially when compared to the number of prostate and breast cancer trials [20, 31]. There is a declining trend in BT usage as part of the standard treatment of CCa, showing a decrease from 83 % in 1988 to 58 % in 2009 [25]. One possible explanation may be the introduction of highly conformal EBRT. Furthermore the limited interest could be a result of the low incidence of advanced CCa in developed countries, where the necessary equipment, infrastructure and funds are available. This leads to slower patient accrual and limited interest from the scientific community. Most registered CCa trials evaluate the different chemotherapeutical approaches where radiotherapy is only part of the standard treatment. Trials concentrated on BT alone are early phase, evaluating mostly different forms of MRI or PET image guidance with toxicity evaluation as primary endpoints (NCT00938106, NCT01399658 and NCT01899404).

It seems that the utilization of newer techniques in form of image guided BT (IGBT) results in a significant improvement in local control of up to 95 % [32–36]. These results are currently under evaluation through the EMBRACE trial (NCT00920920). EMBRACE is an observational clinical study aiming to recruit more than 600 patients. Primary endpoint is local control and treatment morbidity at 5 years. The initial results are already available, with reports of toxicity and quality of life issues [37–39]. The final results in regard to local control and survival are still pending.

Evaluating the toxicities of the combined treatment with EBRT and BT is of special concern. The contribution of BT to healthy tissue total dose is hard to measure. Latest reports regarding utilization of IGBT combined with 3D conformal EBRT show discouraging results regarding quality of life (QOL) [40]. This suggests that higher conformity of IGBT alone has no benefit in terms of QOL and further evaluation as well as optimization of the combined approach is needed.

Data from more conformal EBRT (IMRT, VMAT, helical) combined with IGBT are limited. The possibility of EBRT dose escalation for the primary tumor, parametrial as well as for lymphonodal disease is well described in the literature [41, 42]. Dose escalated EBRT for treating local disease may be an option, especially in institutions lacking the required equipment or experience for conducting IGBT.

EBRT may help in covering parametrial disease, lymphonodal disease or high-risk tumor area not accessible without needle insertion. Such a concept is currently being prospectively evaluated through a phase I/II trial - NCT01793701. Nevertheless, the authors do not suggest that EBRT could replace BT completely.

It is currently unclear which treatment modality (EBRT or BT) contributes more to the applied dosage to organs at risk and consequently to treatment induced toxicity. The combined dose distribution is hard to measure.

#### Endometrial cancer (ECa)

ECa is the most common gynecological malignancy in the western world with most cases being diagnosed in an early stage [43]. The current role of adjuvant RT in the treatment of endometrial cancer is well described in the ASTRO evidence based guidelines [44]. Sorbe et al [45] compared intravaginal BT vs surgery alone for low risk endometrial cancer on 645 patients. The influence on loco-regional recurrence was limited, with OS and overall recurrence rate similar in both researched groups [45]. In its early stages endometrial cancer not receiving adjuvant RT most commonly recurs in the area of the vaginal cuff [46]. The role of postoperative RT for high-intermediate risk endometrial cancer in patients older than 60 years was determined through the PORTEC-2 trial (NCT00376844). The trial shows similar results between EBRT and BT alone for patients with intermediate risk grade 1 disease. However, the results cannot be applied to grade 2 and 3 disease due to the limited number of patients in the trial. Similar results in medium risk endometrial cancer were reproduced by Sorbe B et al [47], where the combined EBRT and BT shows no difference in survival but a significantly higher toxicity [47]. The value of adjuvant therapy and the best treatment modality for high risk stage I and stage II disease should be clarified through the GOG-0249 Trial (NCT00807768). This is a randomized phase III trial comparing adjuvant 3D or IMRT (25–28 fractions) versus HDR or LDR BT combined with carboplatin/paclitaxel chemotherapy. Primary endpoint is recurrence free survival with a planned total accrual number of 562 patients and an estimated final data collection date for the primary outcome measure in March 2013. Results were presented in the form of an abstract on Annual Meeting of the Society of Gynecologic Oncology (SGO) in Tampa 2014. Both concepts appear to be equivalent with slightly more acute toxicities in the chemotherapy arm [48].

EBRT seems to improve survival in some patients. EBRT is recommended in cases of nodal positivity, involved serosa, parametria or adjacent organs. The question that remains is whether or not an additional BT boost is required. It seems that the incidence of vaginal recurrence after pelvic RT is low being between 1.6 and 2.3 % for early stage intermediate or high risk disease [46, 49]. There are currently no randomized trials comparing EBRT vs EBRT with BT. A SEER database analysis suggests that there is a better outcome when a BT boost is applied [50].

#### Breast cancer (BCa)

Most trials involving BT procedures focus on partial breast irradiation (PBI) as an alternative to whole breast irradiation (WBI). Notable phase II trials are the NCT00392184 and NCT00977275 whose results provide a solid basis for clinicians [51–55].

A randomized phase III trial that will hopefully provide new insights into PBI is registered under NCT00103181 (A Randomized Phase III Study of Conventional WBI Versus PBI for Stage 0, I, or II BCa). The trial, initiated by NSABP, and started in March 2005 with a planned total accrual number of 4216 patients. Overall survival and quality of life are the primary endpoints. However BT was not in focus and the allowed BT techniques were Mammosite or single catheter device. Published works from this trial are mostly concentrated on EBRT issues [56–58].

More important from a BT perspective is the phase III trial conducted by the Breast Cancer Working Group of the GEC-ESTRO with the sponsorship of the German Cancer Aid, including 16 centers from 7 European countries and co-chaired by Erlangen and Budapest, the GEC-ESTRO APBI Trial (NCT00402519). The recently published 5-year results showed that adjuvant accelerated partial breast irradiation (APBI) using multicatheter brachytherapy is not inferior to adjuvant WBI with respect to 5-year local control, disease-free survival, and overall survival [59].

No trials directly comparing different BT approaches in terms of application technique or fractionation (e.g., multi-catheter vs. balloon technique; single fraction vs. multiple fractions) were detected. It remains unclear, whether a multicatheter interstitial BT has a superior cosmetic outcome, indeed the required large sample size and the long follow-up makes conducting of such a trial difficult. Furthermore, trials evaluating BT as an alternative to EBRT boost for patients with high-risk BCa would be of interest especially if a dose higher than 16 Gy is required. A retrospective trial published by Knauerhase et al. suggests that the usage of BT may be beneficial in terms of local control [60].

#### Prostate cancer (PCa)

PCa is the tumor entity with the most current trials. There is literature on PCa BT trial results from a time prior to the era of trial registration, but that would exceed the scope of this article. The main therapeutic modalities of BT for PCa can be divided in LDR and HDR, which can be used as a single modality or combined with EBRT.

Grimm et al. [61] retrospectively analyzed PCa series published during 2000–2010 including different treatment modalities. Bearing in mind the potential biases that are intrinsic of observational cohorts and problems in directly comparing different treatment modalities, BT provided the best biochemical control rates as compared to other modalities for low-risk disease. For intermediate-risk disease, the combination of EBRT and BT appeared equivalent to BT alone and superior to EBRT or surgery. For high-risk patients, combination therapies involving EBRT and BT with or without androgen deprivation therapy (ADT) appear superior to more localized treatments such as seed implants, surgery or EBRT alone.

Probably the first studies in prostate BT were published by the Seattle group. Fifteen-year biochemical relapse-free survival (BRFS) and cause-specific survival (CSS) exceeded 80 % for localized PCa treated with I^125^ BT as monotherapy [62]. Excellent results were also seen using a combination of EBRT and a BT boost with seeds in different risk groups [63].

In 1995 Martinez et al. began the HDR monotherapy program at William Beaumont Hospital for low/intermediate risk patients [64], from multi-fraction (4 fractions of 9.5 Gy) to a single fraction of 19 Gy. He used a α/β ratio of 1.5 to give a BED of approximately 260 Gy. He compared the results of LDR BT with HDR. At 5 years, there were no differences in overall survival, in CSS or in BRFS between both groups. There were less chronic urinary side-effects in the HDR group as well as lower sexual impotency after treatment.

Tselis et al. has published his experience on HDR monotherapy for localized PCa, 4 fractions of 9.5 Gy in two implants [65] with good results. However, treatment toxicity could be an issue [66].

Galalae et al. used HDR as a boost to EBRT [67]. Long-term outcomes are presented in three prospective trials in three hospitals: Kiel, William Beaumont and Seattle. He concludes that EBRT with HDR-BT produced excellent long-term outcomes in terms of BRFS, DFS, and CSS.

Also Vargas and Martinez published their results of pelvic EBRT with HDR boost for intermediate and high-risk PCa [68]. HDR dose fractionation increased progressively and was divided into two dose levels. The mean prostate biologic equivalency dose was 88.2 Gy for the low-dose group and 116.8 Gy for the high-dose group (α/β = 1.2). They concluded that dose escalation improved loco-regional control and experienced less biochemical and clinical failures at 5 years.

EBRT followed by BT boost is emerging as an effective approach for unfavorable-risk PCa after the results of the ASCENDE-RT trial. A phase II/III trial that accrued 400 patients with high- and intermediate-risk disease. After receiving whole-pelvis EBRT with 46Gy in 23 fractions, patients underwent random assignment to an I^125^ LDR-BT boost or to a EBRT boost (32 Gy in 16 fractions). Both arms received ADT for 12 months. BPFS rates between the LDR-BT and EBRT boost arms were 83.3 versus 62.4 % at 9 years. However LDR-BT was associated with a significantly greater cumulative incidence of late grade 3 genitourinary toxicity compared with EBRT boost (19 vs. 5 %; *p* < 0.001).

Currently trending is focal radiotherapy in early PCa. Kovács wrote an excellent review on the topic [69]. Cosset published his results using focal I^125^ seeds for selected low-risk patients [70]. Focal treatment using BT is technically feasible however further investigation is needed in order to assess its definite role in PCa.

Tsikkinis et al [71] has shown that despite a strong ongoing clinical research activity in PCa, only 7 out of 123 ongoing unpublished phase 3 trials are evaluating BT alone or BT combined with EBRT (NCT01717677, NCT01936883, NCT00175396, NCT00063882, NCT00247312, and NCT01839994). This corresponds to published findings from the SEER Database [72]. The German PREFERE Trial is a large prospective multicenter trial developed to compare the four possible treatment options (active surveillance, surgery, EBRT and BT) in newly diagnosed low- or early intermediate-risk patients. In total 7600 patients are planned to be recruited and final primary outcome measure is predicted for 2029.

#### Esophageal cancer (ECa)

ECa remains a tumor entity in need for further therapy optimization. Combined modality treatments did not significantly improve the overall outcome of patients with advanced disease. The standard EBRT dose of about 50 Gy [73] may be insufficient for effective local control. Delivering a higher total dose with EBRT is limited due to its proximity to vital organs (lung, heart, and spinal cord) and feared toxicity as well as organ movement and tumor CTV both requiring broader planning margins as compensation [74, 75]. Although a multi-institutional randomized trial of EBRT with vs without additional BT boost showed an improved survival in the BT arm, the proposed concept was not broadly accepted [76]. The benefit was higher in patients with tumors up to 5 cm where the 5-year cause specific survival was 64 %. The mentioned trials were conducted prior to the era of IMRT and IGBT. The trial results were confirmed through an additional prospective trial from Brunner et [77]. Current BT trials for ECa are focusing on palliation.

### Head and neck cancer

The field where BT has the greatest potential is head and neck cancer, either in early stage tumors or as a boost for locally advanced disease. However, we identified only 4 trials that included BT in the treatment protocol and in 2 of them BT was only optional. In one trial the BT aim was palliation (NCT01086488) and the second evaluated I^125^ seed therapy for salivary gland cancer (NCT02048254). The role of BT in squamous cell cancer of the head and neck region is currently not being investigated in any of the identified trials.

### Non-malignant diseases

BT plays also a role in non-malignant diseases. An interesting application of BT is in the treatment of pterygia, a disease most commonly surgically treated with mostly unsatisfactory results. The safety and efficacy of BT as a treatment modality was confirmed through a prospective phase II trial [78]. The mentioned trial was conducted prior to the era of obligatory registration. Further intriguing applications of BT is in the treatment of macular degeneration (evaluated through NCT00100087 and NCT01006538), renal denervation (NCT01968785) and inoperable pituitary macroadenomas (NCT01444209).

Brachytherapy usage is by no means limited to the above-mentioned tumor entities; well-established brachytherapy applications as well as other less frequent indications have been developed and successfully applied during recent years. Corresponding clinical trials are provided as supplementary material (Additional file 1).

#### Limitations

Our analysis is not without limitations. Not every clinical trial is registered on ClinicalTrials.gov; other internationally accepted registries do exist and some trials could be registered elsewhere. Nevertheless, as ClinicalTrials.gov is the largest trials registry available, with 52 % of all registered trials non-US-based, our findings provide a representative, if not complete, picture of current research. Moreover, we cannot exclude the possibility of errors whereby certain trials were not captured for the analysis and/or were misclassified during the selection process. In addition, the data sets for all trials in the database are not always complete and up to date. Another limitation is the high percentage of non-structured data submitted in the form of free text. The sheer data volume and weekly growth rate make a manual trial classification challenging. The problem of classifying oncology related textual information is described in work by Spasic et al. [31].

Despite these limitations we believe our analysis is both unique and important and can provide a realistic snapshot of interventional clinical trials using BT.

Even though, RT is a popular field among clinical scientists and there is a documented increased interest during recent years [79], this does not apply to BT. It seems that this important discipline is being neglected in academic circles and clinics as well. BT requires close patient contact and is a hard and demanding discipline. This appears to discourage rather than motivate the current generation of radiation oncologists.

In this work we analyzed the current status of BT research and demonstrated that several important questions are still open. We believe that there is a lack of interest in BT by the medical community and, as a result, research funding is also very limited. Several important clinical questions still remain but, unfortunately, appropriate answers may not come in the foreseeable future.

## Conclusion

In view of the overall incidence of diseases in which BT has a critical role in the curative treatment, along with several remaining clinical uncertainties, interventional clinical trials evaluating BT procedures appear to be rare.

## Appendix

### Algorithm for classification of source of funding

Basically we decided that money is coming from five different sources:

1. Collaborative Groups
2. Industry
3. NIH
4. Academy
5. A combined public/private funding

From the clinicaltrials.gov database we have two kinds of data registered, the primary sponsor and the collaborators, based on this we identified and assigned the most probable source of funding for each trial.

The rules used were the following:

1. Trials that had no collaborators were assigned based on the primary sponsor alone.
2. If the primary sponsor was an academic institution and the collaborator/s was coming from the industry, Coll. Group or NIH, then the trial was assigned according to the collaborator.
3. If an industry source was involved either as primary sponsor or collaborator and the NIH or a collaborative group as well, then the trial was assigned as combined public/private funding
4. If no industry source was involved and the NIH with a collaborative group were, either as primary sponsor or collaborator, then the trial was assigned as NIH funded.

## Additional file

Additional file 1: Brachytherapy Trials grouped according to trial phase (n: 145) (DOCX 59 kb)

## Abbreviations

3D: three dimensional
ADT: androgen deprivation therapy
APBI: accelerated partial breast irradiation
BCa: breast cancer
BRFS: biochemical relapse-free survival
BT: brachytherapy
CCa: cervical cancer
CSS: cause-specific survival
DFS: disease free survival
EBRT: external beam radiotherapy
ECa: esophageal cancer
EU: European Union
GEC-ESTRO: Groupe Européen de Curiethérapie-European Society for radiotherapy and oncology
HDR: high dose rate
IGBT: image guided brachytherapy
IMRT: intensity-modulated radiation therapy
LDR: low dose rate
MRI: magnetic resonance imaging
NSABP: National Surgical Adjuvant Breast and Bowel Project
PBI: partial breast irradiation
PCa: prostate cancer
PET: positron emissions tomography
QOL: quality of life
RT: radiotherapy
SEER: surveillance, epidemiology, and end results program
US: United States
VMAT: volumetric modulated arc therapy
WBI: whole breast irradiation
