# Supplementary material for: Portfolio of prospective clinical trials including brachytherapy: an analysis of the ClinicalTrials.gov database
Source: Radiat Oncol. 2016 Mar 22;11:48. doi: 10.1186/s13014-016-0624-8 (PMC4804566; doi:10.1186/s13014-016-0624-8)
Supplement: Additional file 1: — Brachytherapy Trials grouped according to trial phase (n: 145) (DOCX 59 kb) [file 13014_2016_624_MOESM1_ESM.docx]

| Additional file 1: Brachytherapy Trials grouped according to trial phase (n: 145) | | | | |
| --- | --- | --- | --- | --- |
| **Phase 4 (n: 13)** | | | | |
| **Nr.** | **ClinicalTrials.gov** | **Short Name** | **Trial  Start Date (D/M/Y)** | **Planned Accrual** |
|  | NCT00046969 | Epoetin Beta in Treating Anemia in Patients With Cervical Cancer | 15/07/02 | 450 |
|  | NCT00180583 | Vision II: Evaluation of GALILEO Intravascular Radiotherapy System | 15/02/02 | 268 |
|  | NCT00714545 | SCRIPPS V: Intracoronary Brachytherapy for Recurrent Restenosis After Multiple Drug-Eluting Stents | 15/04/06 | 60 |
|  | NCT00719017 | Upper Vaginectomy Versus Brachytherapy in Patients With Early Stage Endometrial Cancer Treated With Laparoscopic Surgery | 15/09/07 | 24 |
|  | NCT00742222 | Electronic Xoft Intersociety Brachytherapy Trial: Electronic Brachytherapy (EBT) For Treatment of Early Stage Breast Cancer | 15/05/08 | 63 |
|  | NCT00805701 | Study Assessing The Efficacy And Safety Of Avodart (Dutasteride) At Improving Urinary Symptoms In Men With Prostate Cancer Who Are Undergoing Seed Implantation | 15/01/09 | 40 |
|  | NCT00882089 | Use of the Conturaâ„¢ Catheter in Intermediate-Risk, Pathological Stage 0, I, or II (Up to 3.0 cm) Breast Cancer Patients | 15/10/07 | 13 |
|  | NCT01006538 | Macular EpiRetinal Brachytherapy Versus Lucentis® Only Treatment (MERLOT) | 15/11/09 | 363 |
|  | NCT01017549 | Post Market Study Using the Xoft Axxent System | 15/03/07 | 44 |
|  | NCT01379742 | Comparison of Between ThinSeed and OncoSeed for Permanent Prostate Brachytherapy | 15/04/10 | 240 |
|  | NCT01448447 | Phase IV Trial to Evaluate Breast Brachytherapy Using the Mammosite | 15/12/09 | 200 |
|  | NCT01644669 | Safety and Efficacy Study of the Xoft IORT System | 15/05/12 | 1000 |
|  | NCT01757158 | Cesium-131 Seed Brachytherapy Plus Subtotal Resection for Lung Cancer Patients | 15/02/12 | 40 |
| **Phase 3 (n: 55)** | | | | |
| **Nr.** | **ClinicalTrials.gov** | **Short Name** | **Trial  Start Date (D/M/Y)** | **Planned Accrual** |
|  | NCT00000124 | Collaborative Ocular Melanoma Study (COMS) | 15/11/86 | NR |
|  | NCT00002507 | Radiation Therapy and Chemotherapy in Treating Patients With Head and Neck Cancer | 15/11/92 | NR |
|  | NCT00002884 | Chemotherapy and Radiation Therapy in Treating Patients With Cancer of the Esophagus | 15/03/96 | 326 |
|  | NCT00002898 | Surgery Followed by Chemotherapy in Treating Young Patients With Soft Tissue Sarcoma | 15/01/95 | 400 |
|  | NCT00003078 | Radiation Therapy With or Without Cisplatin or Fluorouracil in Treating Patients With Cancer of the Cervix | 15/10/97 | 870 |
|  | NCT00003749 | Surgery With or Without Lymphadenectomy and Radiation Therapy in Treating Endometrial Cancer | 15/04/98 | 2300 |
| **Nr.** | **ClinicalTrials.gov** | **Short Name** | **Trial  Start Date (D/M/Y)** | **Planned Accrual** |
|  | NCT00006734 | Comparison of Combination Chemotherapy Regimens in Treating Patients With Ewing's Sarcoma or Neuroectodermal Tumor | 15/05/01 | 587 |
|  | NCT00017004 | Radiation Therapy and Cisplatin With or Without Epoetin Alfa in Treating Patients With Cervical Cancer and Anemia | 15/08/01 | 114 |
|  | NCT00023686 | Surgery Versus Internal Radiation in Treating Patients With Stage II Prostate Cancer | 15/10/01 | NR |
|  | NCT00039338 | Chemotherapy Followed By Surgery Vs Radiotherapy Plus Chemotherapy in Patients With Stage IB or II Cervical Cancer | 15/03/02 | 686 |
|  | NCT00063882 | Interstitial Brachytherapy With or Without External-Beam Radiation Therapy in Treating Patients With Prostate Cancer | 15/06/03 | 588 |
|  | NCT00103181 | Radiation Therapy (WBI Versus PBI) in Treating Women Who Have Undergone Surgery For Ductal Carcinoma In Situ or Stage I or Stage II Breast Cancer | 15/03/05 | 4216 |
|  | NCT00107172 | Surgery With or Without Internal Radiation Therapy in Treating Patients With Stage I Non-Small Cell Lung Cancer | 15/07/05 | 226 |
|  | NCT00122746 | Clinical and Experimental Studies to Improve Radiotherapy Outcome in AIDS Cancer Patients | 15/12/04 | 322 |
|  | NCT00122772 | CRP on Radiobiological and Clinical Studies on Viral-Induced Cancer's Response to Radiotherapy | 15/11/05 | 601 |
|  | NCT00142506 | Study of Sildenafil Citrate During and After Radiotherapy/Hormone Therapy for Erectile Function Versus Radiotherapy/Hormone Therapy for Prostate Cancer | 15/02/05 | 290 |
|  | NCT00152919 | Mobicox: Study of Meloxicam to Reduce Prostate Swelling in Permanent Seed Prostate Brachytherapy | 15/02/04 | 100 |
|  | NCT00175396 | Androgen Suppression Combined With Elective Nodal and Dose Escalated Radiation Therapy | 15/05/04 | 400 |
|  | NCT00191100 | Comparative Study of Gemcitabine,Cisplatin and Radiation Versus Cisplatin and Radiation in Cancer of the Cervix | 15/05/02 | 515 |
|  | NCT00193739 | Neoadjuvant Chemotherapy Followed by Surgery Versus Concurrent Chemoradiation in Carcinoma of the Cervix | 15/09/03 | 730 |
|  | NCT00193830 | High Dose Rate (HDR) Versus Low Dose Rate (LDR) Brachytherapy in Carcinoma Cervix | 15/05/96 | 750 |
|  | NCT00201630 | Prophylactic Vs. Therapeutic Use of Uroxatrol in Men Undergoing Brachytherapy | 15/09/05 | 150 |
|  | NCT00231257 | Sirolimus-Eluting Stent vs. Intravascular Brachytherapy in In-Stent Restenotic Coronary Artery Lesions(SISR) | 15/02/03 | 384 |
|  | NCT00241384 | Low Dose Supplemental External Radiation With Pd-103 Versus Pd-103 Alone for Prostate Cancer | 15/01/05 | 300 |
|  | NCT00243646 | Implant and External Radiation for Prostate Cancer With or Without Hormonal Therapy: A Prospective Randomized Trial | 15/08/04 | 6 |
|  | NCT00244309 | Study of Tamsulosin and/or Dutasteride to Relieve Urinary Symptoms After Brachytherapy for Localized Prostate Cancer | 15/11/05 | 348 |
| **Nr.** | **ClinicalTrials.gov** | **Short Name** | **Trial  Start Date (D/M/Y)** | **Planned Accrual** |
|  | NCT00247312 | Pd-103 Dose De-Escalation for Early Stage Prostate Cancer: A Prospective Randomized Trial | 15/10/05 | 600 |
|  | NCT00376844 | External-Beam Radiation Therapy or Implant Radiation Therapy Compared With Observation in Treating Patients Who Have Undergone Surgery for Stage I Endometrial Cancer | 15/05/06 | 104 |
|  | NCT00402519 | APBI Versus EBRT Therapy After Breast Conserving Surgery for Low-risk Breast Cancer | 15/11/04 | 1300 |
|  | NCT00411138 | Randomized Trial of Radiation Therapy With or Without Chemotherapy for Endometrial Cancer | 15/10/06 | 670 |
|  | NCT00486499 | I-125 Versus Pd-103 for Medium Risk Prostate Cancer | 15/03/03 | 660 |
|  | NCT00494039 | I-125 Versus Pd-103 for Low Risk Prostate Cancer | 15/01/98 | 602 |
|  | NCT00494546 | High Versus Low Dose Supplemental External Radiation With Pd-103 for Prostate Cancer | 15/01/99 | 568 |
|  | NCT00644618 | Randomized Controlled Trial of Gemcitabine Combined With 125I Brachytherapy | 15/01/03 | 120 |
|  | NCT00664456 | Luteinizing Hormone-Releasing Hormone Agonist Therapy and Iodine I 125 Implant in Treating Patients With Previously Untreated Prostate Cancer | 15/04/08 | 420 |
|  | NCT00665197 | Palliative Radiotherapy and Brachytherapy for Oesophageal Cancer Dysphagia | 15/02/07 | 200 |
|  | NCT00807768 | Pelvic Radiation Therapy or Vaginal Implant Radiation Therapy, Paclitaxel, and Carboplatin in Treating Patients With High-Risk Stage I or Stage II Endometrial Cancer | 15/03/09 | 562 |
|  | NCT00929591 | SWOG-8814 Tamoxifen With or Without Combination Chemotherapy in Postmenopausal Women Who Have Undergone Surgery for Breast Cancer | 15/05/89 | 1558 |
|  | NCT00942357 | Carboplatin and Paclitaxel With or Without Cisplatin and Radiation Therapy in Treating Patients With Stage I, Stage II, Stage III, or Stage IVA Endometrial Cancer | 15/06/09 | 804 |
|  | NCT01054274 | a Multicentric Randomized Controlled Trial of Self-Expandable Esophageal Radiation Stent | 15/12/09 | 180 |
|  | NCT01174017 | Ability of a New Design of Iodine 125 Seed to Maintain Intended Position When Implanted in the Prostate | 15/09/10 | 41 |
|  | NCT01336894 | Surgery With or Without Internal Radiation Therapy Compared With Stereotactic Body Radiation Therapy in Treating Patients With High-Risk Stage I Non-Small Cell Lung Cancer | 15/05/11 | 420 |
|  | NCT01351116 | A Trial to Evaluate the Improvement in Lung Cancer Patients Receiving Radiation With or Without Brachytherapy | 15/10/11 | 250 |
|  | NCT01414608 | Cisplatin and Radiation Therapy With or Without Carboplatin and Paclitaxel in Patients With Locally Advanced Cervical Cancer | 15/01/12 | 780 |
|  | NCT01474356 | Hyperthermia Combined Brachytherapy in CCU | 15/11/06 | 224 |
|  | NCT01561586 | Tri-weekly Cisplatin Based Chemoradiation in Locally Advanced Cervical Cancer | 15/03/12 | 590 |
|  | NCT01566240 | Induction Chemotherapy Plus Chemoradiation as First Line Treatment for Locally Advanced Cervical Cancer | 15/09/12 | 770 |
| **Nr.** | **ClinicalTrials.gov** | **Short Name** | **Trial  Start Date (D/M/Y)** | **Planned Accrual** |
|  | NCT01717729 | Hepatocellular Carcinoma Treated With Iodine-125 Implantation | 15/01/00 | 136 |
|  | NCT01820858 | The Efficacy and Safety of the Postoperative Adjuvant Treatment in Patients With High-risk Stage I Endometrial Carcinoma | 15/11/12 | 300 |
|  | NCT01839994 | Conformal Radiotherapy (CRT) Alone Versus CRT Combined With HDR BT or Stereotactic Body Radiotherapy for Prostate Cancer | 15/06/13 | 350 |
|  | NCT01917695 | Study on Early Stage Bulky Cervical Cancers | 15/08/13 | 180 |
|  | NCT01936883 | Improving Quality of Life After Prostate Brachytherapy: a Comparison of HDR and LDR Brachytherapy | 15/01/14 | 200 |
|  | NCT02001779 | IRS(Irradiation Stent) vs. CS(Conventional Stent) Insertion in Inoperable Malignant Biliary Obstruction | 15/10/13 | 238 |
|  | NCT02048254 | Study of I-125 Brachytherapy Versus Intensity-modulated Radiation Therapy to Treat Inoperable Salivary Gland Cancer | 15/02/14 | 90 |
|  | NCT02303327 | Comparative Study of Radiotherapy Treatments to Treat High Risk Prostate Cancer Patients | 15/01/15 | 296 |
| **Phase 2/3 (n: 4)** | | | | |
| **Nr.** | **ClinicalTrials.gov** | **Short Name** | **Trial  Start Date (D/M/Y)** | **Planned Accrual** |
|  | NCT00160875 | Pre-operative Chemo (CPT11, Cisplatin), Radiotherapy, Plus Surgery for Resectable Esophageal Cancer | 15/04/09 | 54 |
|  | NCT00287573 | Randomized Trial Evaluating Slow-Release Formulation TAXUS Paclitaxel-Eluting Coronary Stent in the Treatment of In-Stent Restenosis | 15/06/03 | 488 |
|  | NCT01786278 | Comparison Study of Brachytherapy and Endoscopic Stenting for Dysphagia in Esophago-Gastric Junction Cancer | 15/02/13 | 80 |
|  | NCT02258087 | HDR vs LDR Brachytherapy as Monotherapy in the Treatment of Localized Prostate Cancer. | 15/09/14 | 50 |
| **Phase 2 (n: 79)** | | | | |
| **Nr.** | **ClinicalTrials.gov** | **Short Name** | **Trial  Start Date (D/M/Y)** | **Planned Accrual** |
|  | NCT00002492 | Combination Chemotherapy in Treating Patients With Soft Tissue Sarcoma | 15/11/91 | NR |
|  | NCT00002689 | Radiation Therapy Plus Chemotherapy in Treating Patients With Pancreatic Cancer | 15/09/95 | 48 |
|  | NCT00002791 | Chemotherapy Plus Radiation Therapy Followed by Surgery in Treating Patients With Soft Tissue Sarcoma | 15/02/97 | NR |
|  | NCT00002804 | Combination Chemotherapy, Surgery, and Radiation Therapy in Treating Children With Advanced Soft Tissue Sarcoma | 15/09/96 | 43 |
|  | NCT00003312 | Brachytherapy in Treating Patients With Prostate Cancer | 15/09/98 | 95 |
|  | NCT00003574 | Radiation Therapy in Treating Patients With Progressive or Recurrent Malignant Brain Tumors | 15/04/99 | NR |
| **Nr.** | **ClinicalTrials.gov** | **Short Name** | **Trial  Start Date (D/M/Y)** | **Planned Accrual** |
|  | NCT00006359 | Androgen Suppression Plus Radiation Therapy in Treating Patients With Prostate Cancer | 15/09/00 | 63 |
|  | NCT00006365 | External-Beam Radiation Therapy Plus Implanted Radiation Therapy in Treating Patients With Prostate Cancer | 15/11/00 | 138 |
|  | NCT00008112 | Cisplatin Combined With Radiation Therapy and Hyperthermia in Treating Patients With Stage II, Stage III, or Stage IV Cervical Cancer | 15/06/00 | NR |
|  | NCT00032006 | Hormone Therapy Followed By Internal Radiation Therapy in Treating Patients With Locally Recurrent Prostate Cancer | 15/02/02 | NR |
|  | NCT00039624 | Magnetic Resonance-Guided High-Dose Brachytherapy (Short-Range Radiation Therapy) for Prostate Cancer | 15/05/02 | 12 |
|  | NCT00078923 | Soy Isoflavones in Treating Patients Who Are Undergoing Radical Prostatectomy for Stage I or Stage II Adenocarcinoma of the Prostate | 15/11/01 | 32 |
|  | NCT00086736 | Neoadjuvant Eflornithine and Bicalutamide Compared With Eflornithine Alone, Bicalutamide Alone, and No Neoadjuvant Therapy in Treating Patients With Localized Prostate Cancer Undergoing Brachytherapy or Radical Prostatectomy | 15/11/01 | 34 |
|  | NCT00091390 | Radiation Therapy in Treating Patients With Stage II or Stage III Prostate Cancer | 15/07/04 | 129 |
|  | NCT00278304 | Radiation Therapy in Treating Patients With Cervical Cancer | 15/09/05 | 20 |
|  | NCT00369122 | Bevacizumab, Radiation Therapy, and Cisplatin in Treating Patients With Previously Untreated Locally Advanced Cervical Cancer | 15/08/06 | 60 |
|  | NCT00379665 | Intratumoral Cancer Chemotherapy Through a Flexible Bronchoscope as an Adjunct to Brachytherapy | 15/10/05 | 25 |
|  | NCT00392184 | Partial Breast Irradiation With Multi-Catheter Brachytherapy for pT1-2pN0 Breast Cancer After Breast Conserving Surgery | 15/11/00 | NR |
|  | NCT00404326 | Hydralazine and Valproate Plus Cisplatin Chemoradiation in Cervical Cancer | 15/05/05 | 18 |
|  | NCT00450411 | Ultrasound-Guided Implant Radiation Therapy in Treating Patients With Locally Recurrent Prostate Cancer Previously Treated With External-Beam Radiation Therapy | 15/05/07 | 100 |
|  | NCT00462397 | Paclitaxel and Carboplatin Followed by Cisplatin and Radiation Therapy in Treating Patients With Stage IB, Stage II, Stage III, or Stage IVA Cervical Cancer | 15/06/05 | 50 |
|  | NCT00477841 | Self-Expandable Esophageal Radiation Stent:a Randomized Controlled Trial in Patients With Advanced Esophageal Cancer | 15/04/04 | 60 |
|  | NCT00492778 | Radiation Therapy With or Without Cisplatin in Treating Patients With Recurrent Endometrial Cancer | 15/02/08 | 164 |
|  | NCT00499057 | Partial Breast Irradiation With Interstitial High Dose Rate Brachytherapy | 15/08/03 | 150 |
|  | NCT00525720 | Brachytherapy for Prostatic Carcinoma Patients | 15/08/06 | 300 |
|  | NCT00542490 | Vaginal Cuff Brachytherapy Followed by Chemotherapy in Patients With Endometrioid Cancer | 15/09/07 | 120 |
| **Nr.** | **ClinicalTrials.gov** | **Short Name** | **Trial  Start Date (D/M/Y)** | **Planned Accrual** |
|  | NCT00611624 | Mammosite Breast Brachytherapy Optimization in the Treatment of Breast Carcinoma | 15/05/06 | 40 |
|  | NCT00684905 | Leuprolide, Bicalutamide, and Implant Radiation Therapy in Treating Patients With Locally Recurrent Prostate Cancer After External-Beam Radiation Therapy | 15/04/00 | 50 |
|  | NCT00794339 | Copper Cu 64-ATSM and PET/CT Scan in Predicting Disease Progression in Patients With Newly-Diagnosed Stage IB, Stage II, Stage III, or Stage IVA Cervical Cancer Who Are Undergoing Chemoradiotherapy Per NCCN Guidelines | 15/07/09 | 73 |
|  | NCT00807300 | Computed Tomography (CT) - Guided Brachytherapy Versus Transarterial Chemoembolization in Patients With Unresectable Hepatocellular Carcinoma | 15/10/06 | 68 |
|  | NCT00866554 | Efficacy and Toxicity of Bicalutamide and Dutasteride vs. Standard Care for Prostate Cytoreduction for Brachytherapy | 15/03/09 | 88 |
|  | NCT00916500 | Concurrent Chemoradiation With Cisplatin Every 3 Week in Advanced Cervical Cancer | 15/03/06 | 71 |
|  | NCT00924027 | A Study of Patients Receiving High-Dose Rate Brachytherapy | 15/03/09 | 112 |
|  | NCT00938106 | Optimized Magnetic Resonance Brachytherapy (MR BT) in Cervix Cancer | 15/05/08 | 60 |
|  | NCT00945061 | Radiation Therapy in Treating Patients With Recurrent Breast Cancer | 15/09/08 | 30 |
|  | NCT01008514 | Partial-Breast Radiation Therapy in Treating Women With Early-Stage Breast Cancer | NR | 0 |
|  | NCT01019720 | Internal Radiation Therapy of the Breast in Treating Women With Early-Stage Breast Cancer | 15/11/09 | 22 |
|  | NCT01086488 | Foscan®-Mediated Photodynamic Therapy Versus Brachytherapy in Patients With Nasopharyngeal Carcinoma | 15/01/09 | 66 |
|  | NCT01117402 | Tomotherapy in Postsurgery Recurrent Carcinoma Cervix | 15/12/08 | 90 |
|  | NCT01149304 | Preventive Effect of Enoxaparin, Pentoxifylline and Ursodeoxycholic Acid to Radiation Induced Liver Toxicity | 15/06/09 | 44 |
|  | NCT01158248 | Panitumumab, Cisplatin, and Pelvic Radiation Therapy in Treating Patients With Stage IB, Stage II, or Stage III Cervical Cancer | 15/02/10 | 50 |
|  | NCT01175694 | Dose Optimization for Pulsed-dose-rate (PDR)/High-dose-rate (HDR) Brachytherapy Alone for Early Breast Cancer | 15/01/10 | 200 |
|  | NCT01185145 | Accelerated Partial Breast Radiotherapy With Either Mammosite or Intensity Modulated Radiotherapy | 15/02/04 | 291 |
|  | NCT01226979 | Study of High-Dose-Rate Endorectal Brachytherapy (HDRBT) in the Treatment of Locally Advanced Low Rectal Cancer | 15/09/10 | 30 |
|  | NCT01248741 | CT-validation of Ultrasound Based Planning for High Dose Rate (HDR) Prostate Brachytherapy Using Vitesse | 15/12/10 | 25 |
|  | NCT01274962 | A Study on the Timing of FOLFOX for Patients With Operable, Node Positive Rectal Cancer | 15/11/09 | 180 |
| **Nr.** | **ClinicalTrials.gov** | **Short Name** | **Trial  Start Date (D/M/Y)** | **Planned Accrual** |
|  | NCT01276730 | Advanced Cervical Cancer Trial in India | 15/10/07 | 200 |
|  | NCT01354951 | Assessing the Potential for Reduced Toxicity Using Focal Brachytherapy in Early Stage, Low Volume Prostate Cancer | 15/05/11 | 80 |
|  | NCT01374087 | Study to Assess the Efficacy of Brachytherapy With or Without Hormone Therapy, Using Triptorelin 22.5mg in Patients With Recurrence of Prostate Cancer | 15/11/11 | 37 |
|  | NCT01399658 | Image-Guided Gynecologic Brachytherapy | 15/09/11 | 100 |
|  | NCT01409876 | HistScanning- Based PDR Brachytherapy in Prostate Cancer HistoScanning- Based Interstitial PDR Brachytherapy | 15/09/11 | 100 |
|  | NCT01444209 | Radioactive Iodine Implants for Pan-invasive Pituitary Macroadenomas | 15/10/11 | 24 |
|  | NCT01446991 | Feasibility and Toxicity of Degarelix for Prostate Downsizing Prior to Permanent Seed Prostate Brachytherapy | 15/04/12 | 50 |
|  | NCT01552239 | Preoperative Radiotherapy for Sarcomas of the Extremities With Intensity-Modulation, Image-Guidance and Small Safety-margins | 15/08/11 | 50 |
|  | NCT01594099 | Concurrent Chemoradiotherapy for Cervical Cancer in Elderly Women | 15/04/12 | 45 |
|  | NCT01605097 | High Dose Rate Prostate Brachytherapy: Dose Escalation to Dominant Intra-prostatic Nodule | 15/05/12 | 15 |
|  | NCT01639625 | Concurrent Treatment of Squamous Cell Carcinoma or Adenocarcinoma of the Cervix With CIGB-300 for Local Application | 15/05/11 | 18 |
|  | NCT01659424 | Phase II Trial of Preoperative High-dose-rate Endorectal Brachytherapy and FOLFOX Chemotherapy for Rectal Cancer | 15/06/11 | 0 |
|  | NCT01802242 | Tumor TARGET Prostate Cancer | 15/10/12 | 110 |
|  | NCT01802307 | Focal Therapy for Prostate Cancer | NR | 50 |
|  | NCT01835171 | Cisplatin and Radiation Therapy With or Without Triapine in Treating Patients With Previously Untreated Stage IB-IVA Cervical Cancer or Stage II-IVA Vaginal Cancer | 15/04/13 | 73 |
|  | NCT01851018 | Toxicity Comparison Between Hypofractionated Radiotherapy With HDR Brachytherapy Boost Versus Standard Treatment | 15/05/12 | 30 |
|  | NCT01890096 | A Phase II Trial of High Dose-Rate Brachytherapy as Monotherapy in Low and Intermediate Risk Prostate Cancer | 15/05/13 | 174 |
|  | NCT01898065 | Evaluation of Hypoxia by PET With F-Miso in Radiation Therapy of Prostate Cancer | 15/06/12 | 20 |
|  | NCT01902680 | Phase II Study of Feasibility of Focal Therapy for Prostate Cancer of Good Prognosis With Permanent I125 Localized Implant. | 15/08/13 | 17 |
|  | NCT01909388 | Dose Escalation to Dominant Intraprostatic Lesions (DIL) With MRI-TRUS Fusion High Dose Rate (HDR) Prostate Brachytherapy | 15/06/13 | 15 |
| **Nr.** | **ClinicalTrials.gov** | **Short Name** | **Trial  Start Date (D/M/Y)** | **Planned Accrual** |
|  | NCT01956058 | Brachytherapy for Recurrent Prostate Cancer | 15/09/13 | 28 |
|  | NCT01961531 | BrUOG 291: Five Fraction Partial Breast Irradiation Using Non-invasive Image-guided Breast Brachytherapy (NIBB) | 15/07/14 | 40 |
|  | NCT01973101 | Non-comparative Study of Treatment With Induction Chemotherapy With Cisplatin and Gemcitabine Followed by Chemoradiation or Definitive Chemoradiation in Invasive Locally Advanced Carcinomas of Uterine Cervix. | 15/06/12 | 120 |
|  | NCT01982786 | Feasibility Trial Of Image Guided External Beam Radiotherapy With Or Without High Dose Rate Brachytherapy Boost In Men With Intermediate-Risk Prostate Cancer | 15/11/13 | 60 |
|  | NCT02017704 | Chemoradiation or Brachytherapy for Rectal Cancer | 15/10/13 | 138 |
|  | NCT02077335 | Study of High-dose Rate (HDR) Monotherapy for Low and Intermediate Risk Prostate Cancer | 15/04/14 | 50 |
|  | NCT02091050 | 2D vs 3D Planning for High-Dose Rate (HDR) Gynecological Brachytherapy | 15/06/14 | 60 |
|  | NCT02225925 | Intraoperative Dosimetry for Prostate Brachytherapy Using Fluoroscopy and Ultrasound | 15/08/14 | 24 |
|  | NCT02280356 | Radiation Therapy in Combination With Brachytherapy for Clinically Localized, Intermediate Risk Prostate Cancer | 15/10/14 | 44 |
|  | NCT02283346 | Single Fraction HDR Brachytherapy Plus Hypofractionated EBRT for Low-risk Prostate Cancer: Phase II Trial | 15/11/14 | 60 |
|  | NCT02290366 | Prospective Evaluation of Focal Brachytherapy Using Cesium-131 For Patients With Low Risk Prostate Cancer | 15/11/14 | 100 |
|  | NCT02297672 | Partial Breast Irradiation Using Interstitial Permanent Palladium-103 Seed Implant | 15/01/15 | 25 |
|  | NCT02391051 | Focal Brachytherapy in Patients With Selected "Low-risk" Prostate Cancer - a Phase-II-trial | 15/10/14 | 50 |
| **Phase 1/2 (n: 16)** | | | | |
| **Nr.** | **ClinicalTrials.gov** | **Short Name** | **Trial  Start Date (D/M/Y)** | **Planned Accrual** |
|  | NCT00002873 | Radiation Therapy in Treating Women With Stage I or Stage II Breast Cancer | 15/05/97 | NR |
|  | NCT00003379 | Radiation Therapy Plus Paclitaxel and Cisplatin in Treating Patients With Cervical Cancer | 15/11/99 | 40 |
|  | NCT00005939 | Brachytherapy in Treating Patients With Recurrent Prostate Cancer | 15/11/99 | NR |
|  | NCT00012012 | Radiation Therapy and Cisplatin With or Without Amifostine for Patients With Stage IIIB or IVA Cervical Cancer | 15/08/01 | 45 |
|  | NCT00023660 | Radiation Therapy Plus Celecoxib, Fluorouracil, and Cisplatin in Locally Advanced Cervical Cancer | 15/08/01 | 84 |
|  | NCT00100087 | Safety Study for Treatment of Wet Macular Degeneration Using the TheraSight(TM) Ocular Brachytherapy System | 15/10/04 | 30 |
| **Nr.** | **ClinicalTrials.gov** | **Short Name** | **Trial  Start Date (D/M/Y)** | **Planned Accrual** |
|  | NCT00252941 | Prophylactic Urethral Stenting With Memokath After Prostate Implantation for Prostate Adenocarcinoma | 15/11/05 | 20 |
|  | NCT00510250 | A Phase I/II Study of Cisplatin and Radiation in Combination With Sorafenib in Cervical Cancer | 15/06/07 | 30 |
|  | NCT00977275 | Treatment of T1N0 Invasive Breast Carcinoma by Local Excision Implant | 15/06/97 | 45 |
|  | NCT01227642 | Using Ultrasound Spectrum Analysis (USA) to Guide Dose Escalated Prostate Brachytherapy | 15/02/07 | 15 |
|  | NCT01391065 | MR-PET Guided Biologically Optimised Interstitial Brachytherapy | 15/03/11 | 60 |
|  | NCT01727011 | Single Fraction Elderly Breast Irradiation (SiFEBI) | 15/11/12 | 25 |
|  | NCT01793701 | Intensity-modulated Radiotherapy for Locally Advanced Cervical Cancer | 15/07/10 | 44 |
|  | NCT01913106 | HSV-tk + Valacyclovir Therapy in Combination With Brachytherapy for Recurrent Prostate Cancer | 15/06/07 | 25 |
|  | NCT01913197 | Using Magnetic Resonance Imaging (MRI) to Guide Differential-Dose Prostate Brachytherapy | 15/08/13 | 78 |
|  | NCT02178280 | Safety Study of Liver Transplantation for Hilar Cholangiocarcinoma | 15/05/13 | 60 |
| **Phase 1 (n: 33)** | | | | |
| **Nr.** | **ClinicalTrials.gov** | **Short Name** | **Trial  Start Date (D/M/Y)** | **Planned Accrual** |
|  | NCT00001442 | A Pilot Study of Paclitaxel With Radiation Therapy for Locally Advanced Head and Neck Cancer | 15/07/95 | 35 |
|  | NCT00002949 | Vinorelbine and Paclitaxel Plus Radiation Therapy in Treating Patients With Advanced Cancer Arising in the Pelvis | 15/07/96 | 33 |
|  | NCT00003377 | Radiation Therapy, Paclitaxel, and Cisplatin in Treating Patients With Cancer of the Cervix | 15/11/99 | 29 |
|  | NCT00003876 | Internal Radiation Therapy Plus Carmustine Implants in Treating Patients With Recurrent or Refractory Malignant Glioma | 15/04/99 | NR |
|  | NCT00004129 | Phosphorus 32 in Treating Patients With Glioblastoma Multiforme | 15/09/99 | 12 |
|  | NCT00014066 | Photodynamic Therapy Plus Brachytherapy in Treating Patients With Lung Cancer | 15/03/93 | NR |
|  | NCT00045474 | Brachytherapy in Treating Patients With Recurrent Malignant Glioma | 15/10/02 | NR |
|  | NCT00053183 | Surgery Followed by Radiation Therapy in Treating Patients With Newly Diagnosed Glioblastoma Multiforme | 15/10/03 | NR |
|  | NCT00068549 | Radiation Therapy Plus Cisplatin and Gemcitabine in Treating Patients With Cervical Cancer | 15/10/03 | 13 |
|  | NCT00098995 | Tirapazamine, Cisplatin, and Radiation Therapy in Treating Patients With Stage IB, Stage II, Stage III, or Stage IVA Cervical Cancer | 15/12/04 | 22 |
|  | NCT00104910 | Cetuximab, Cisplatin, and Radiation Therapy in Treating Patients With Stage IB, Stage II, Stage III, or Stage IVA Cervical Cancer | 15/01/05 | 64 |
| **Nr.** | **ClinicalTrials.gov** | **Short Name** | **Trial  Start Date (D/M/Y)** | **Planned Accrual** |
|  | NCT00217516 | Selenium in Treating Patients Who Are Undergoing Brachytherapy for Stage I or Stage II Prostate Cancer | 15/03/05 | 18 |
|  | NCT00243321 | High Dose-Rate Brachytherapy and/or Intensity Modulated External Beam Radiation Therapy for Prostate Cancer | 15/02/03 | 27 |
|  | NCT00287911 | Topotecan, Cisplatin, and Radiation Therapy in Treating Patients With Advanced Cervical Cancer | 15/02/05 | 18 |
|  | NCT00334321 | Pelvic IMRT With Tomotherapy in Post-Hysterectomy Endometrial Cancer Patients | 15/04/06 | 65 |
|  | NCT00335998 | Phase I Study of Intravenous Triapine in Combination With Pelvic Radiation Therapy With or Without Weekly Cisplatin Chemotherapy for Locally Advanced Cervical, Vaginal, or Pelvic Gynecologic Malignancies | 15/03/06 | 24 |
|  | NCT00359866 | Pelvic IMRT With Tomotherapy: A Phase I Feasibility Study in Post-Hysterectomy Cervical Cancer Patients | 15/02/06 | 28 |
|  | NCT00426959 | Study of Image-guided Dosimetry for Interstitial Prostate Brachytherapy | 15/02/05 | 6 |
|  | NCT00448643 | Whole-Abdominal Radiation Therapy and Cisplatin in Treating Patients With Stage III or Stage IV Endometrial Cancer That Has Been Removed by Surgery | 15/05/02 | 12 |
|  | NCT00750399 | Effect of Intravitreal Ranibizumab on Radiation Retinopathy Following Plaque Brachytherapy for Choroidal Melanoma | 15/10/08 | 10 |
|  | NCT00811408 | Cidofovir in Treating Patients With Stage IB, Stage II, Stage III, or Stage IVA Cervical Cancer Who Are Receiving Chemotherapy and Radiation Therapy | 15/04/08 | 24 |
|  | NCT01295502 | Cisplatin and Radiation Therapy Followed by Paclitaxel and Carboplatin in Treating Patients With Stage I, Stage II, Stage III, or Stage IV Cervical Cancer | 15/04/11 | 45 |
|  | NCT01366833 | Optimal Management of Malignant Dysphagia | 15/06/11 | 72 |
|  | NCT01460810 | Study Of Surgical Radiation Shielding With Vitrectomy And Silicone Oil Tamponade For The Protection Of Radiation Induced Ocular Injury | 15/07/11 | 20 |
|  | NCT01485731 | Safety Study of Nelfinavir + Cisplatin + Pelvic Radiation Therapy to Treat Cervical Cancer | 15/01/12 | 24 |
|  | NCT01554410 | Intensity Modulated Radiation Therapy With Cisplatin & Gemcitabine in Locally Advanced Cervical Carcinoma | 15/08/10 | 19 |
|  | NCT01583920 | Focal Salvage HDR Brachytherapy for the Treatment of Prostate Cancer | 15/07/12 | 10 |
|  | NCT01655836 | High-Dose Rate Brachytherapy and Stereotactic Body Radiation Therapy in Treating Patients With Prostate Cancer | 15/10/12 | 42 |
|  | NCT01711515 | Chemoradiation Therapy and Ipilimumab in Treating Patients With Locally Advanced Cervical Cancer | 15/10/12 | 28 |
|  | NCT01958658 | AZD1775, Cisplatin, and Radiation Therapy in Treating Patients With Cervical Cancer | 15/09/13 | 57 |
|  | NCT01991808 | Suitability of DCE-MRI for Detection of Vascular Changes After VBT | 15/10/13 | 20 |
|  | NCT02058550 | Phone or Email Reminder in Increasing Vaginal Dilator Use in Patients With Gynecologic Cancers Undergoing Brachytherapy | 15/09/14 | 72 |
|  | NCT02199236 | Dose Escalation Trial of Endoluminal High-Dose-Rate Brachytherapy With Concurrent Chemotherapy for Rectal or Anal Cancer in Patients With Recurrent Disease or Undergoing Non-Operative Management | 15/07/14 | 24 |
| **Phase N/A (n: 45)** | | | | |
| **Nr.** | **ClinicalTrials.gov** | **Short Name** | **Trial  Start Date (D/M/Y)** | **Planned Accrual** |
|  | NCT00527293 | Partial Breast Radiation Therapy in Treating Women Undergoing Breast-Conserving Therapy for Early Stage Breast Cancer | 15/06/07 | 29 |
|  | NCT00012935 | Treatment Decision Intervention for Veterans With Prostate Cancer | NR | 100 |
|  | NCT00079365 | Fluorouracil, External-Beam Radiation Therapy, and Gemcitabine With or Without Brachytherapy Using Phosphorus P32 in Treating Patients With Locally or Regionally Advanced Unresectable Adenocarcinoma of the Pancreas | 15/05/01 | NR |
|  | NCT00112307 | Magnetic Resonance Imaging Guided Gynecologic Brachytherapy | 15/03/03 | 15 |
|  | NCT00126854 | High Field Magnetic Resonance Spectroscopy Imaging for Follow Up of Prostate Cancer Post Brachytherapy Implantation | 15/10/05 | 10 |
|  | NCT00127816 | Improving Assessment (and Ultimately Outcomes) of Permanent Prostate Implant Therapy | 15/04/05 | 40 |
|  | NCT00185744 | Accelerated Partial Breast Irradiation Following Lumpectomy for Breast Cancer | 15/09/02 | 400 |
|  | NCT00319462 | Localization of Point A in Cervical Cancer | 15/11/06 | 20 |
|  | NCT00381966 | Study of Robotic Template Guidance for Needle Placement in Transperineal Prostate Brachytherapy | 15/06/06 | 5 |
|  | NCT00459849 | Tilting of Radioactive Plaques After Initial Accurate Placement for Treatment of Uveal Melanoma | 15/07/00 | 175 |
|  | NCT00534196 | Implant Radiation Therapy Using Radioactive Iodine in Treating Patients With Localized Prostate Cancer | 15/07/05 | 7000 |
|  | NCT00573833 | Internal Radiation Therapy in Treating Patients With Prostate Cancer | 15/11/07 | 23 |
|  | NCT00593346 | Evaluation of Accelerated Partial Breast Brachytherapy | 15/12/03 | 150 |
|  | NCT00599989 | Partial Breast Radiation Therapy in Treating Women Undergoing Breast Conservation Therapy for Early-Stage Breast Cancer | 15/01/05 | 100 |
|  | NCT00604526 | High Dose Rate Prostate Brachytherapy as Salvage for Locally Recurrent Prostate Cancer Previously Treated With External Beam Radiotherapy | 15/09/06 | 42 |
|  | NCT00662233 | Combination Chemotherapy in Treating Patients With Sarcoma | 15/10/91 | 120 |
|  | NCT00681993 | Trial of Partial Breast Irradiation With Various Concurrent Chemotherapy Regimens | 15/04/08 | 50 |
|  | NCT00770822 | Clinical Study of the Sonablate® 500 to Treat Localized (T1c/T2a) Prostate Cancer | 15/04/07 | 466 |
|  | NCT00826813 | Trial Using 125I Embedded Stent in Patients With Advanced Esophageal Cancer | 15/01/09 | 250 |
|  | NCT00911079 | Pilot Study of a Catheter-based Ultrasound Hyperthermia System | 15/04/09 | 24 |
| **Nr.** | **ClinicalTrials.gov** | **Short Name** | **Trial  Start Date (D/M/Y)** | **Planned Accrual** |
|  | NCT00913939 | High-Dose-Rate Brachytherapy | 15/05/09 | 100 |
|  | NCT00922961 | Research for Predictive Biologic Parameters of Cutaneous Hypersensitivity After Brachytherapy in Breast Cancer | 15/09/08 | 25 |
|  | NCT01016561 | Magnetic Resonance Imaging-Based Radiation Therapy and Cisplatin in Patients With Stage I, Stage II, Stage III, or Stage IV Cervical Cancer | 15/07/09 | 20 |
|  | NCT01045187 | Feasibility Study Using the Xoft System for the Treatment of Endometrial Cancer | 15/10/08 | 15 |
|  | NCT01098331 | Implant Radiation Therapy or Surgery in Treating Patients With Prostate Cancer | 15/05/09 | 400 |
|  | NCT01191164 | Pilot Study of Partial Breast Irradiation Utilizing Permanent Breast Seed Implant to Treat Breast Cancer | 15/09/11 | 5 |
|  | NCT01367301 | Radiation Therapy, Paclitaxel, and Carboplatin in Treating Patients With Uterine Cancer | 15/05/11 | 18 |
|  | NCT01549795 | Liver Transplantation for Hilar Cholangiocarcinoma in Association With Neoadjuvant Radio- and Chemo-therapy | 15/01/12 | 33 |
|  | NCT01601691 | Protection of Rectum From High Radiation Doses | 15/04/12 | 10 |
|  | NCT01663753 | Diagnostic Performance of 18F-FDG-PET and Diffusion-weighted MRI in the Assessment of Stage IB to IIB2 Cervical Squamous-cell Carcinoma Response to Concomitant Radiochemotherapy and Brachytherapy | 15/01/12 | 148 |
|  | NCT01680523 | Radical Hysterectomy Followed by Tailored Adjuvant Therapy Versus Primary Chemoradiation Therapy in Bulky Early-stage Cervical Cancer (KGOG 1029) | 15/09/12 | 409 |
|  | NCT01706705 | Magnetic Resonance Imaging (MRI) Brachytherapy Applicator Study | 15/10/12 | 57 |
|  | NCT01717677 | Evaluation of Four Treatment Modalities in Prostate Cancer With Low or "Early Intermediate" Risk | 15/10/12 | 7600 |
|  | NCT01757717 | Image-Guided Navigation for High Dose Rate Temporary Interstitial Brachytherapy in the Palliative Management of Previously Treated Tumors of the Spine and Pelvis | 15/01/13 | 20 |
|  | NCT01830166 | Focal Therapy for Prostate Cancer - A Pilot Study of Focal Low Dose Rate Brachytherapy | 15/05/13 | 10 |
|  | NCT01851772 | Feasibility Study of the Xoft® Axxent® Electronic Brachytherapy System for the Treatment of Cervical Cancer | 15/11/13 | 5 |
|  | NCT01878058 | Identifying Prostate Brachytherapy Seeds Using MRI | 15/10/12 | 10 |
|  | NCT01899404 | MRI FDG PET Imaging Cervix | 15/10/12 | 30 |
|  | NCT01918605 | Protection of Rectum From High Radiation Doses Using a Spacer | 15/06/13 | 20 |
|  | NCT01958580 | Gemcitabine Hydrochloride, Docetaxel, and Radiation Therapy in Treating Patients With Uterine Sarcoma That Has Been Removed By Surgery | 15/06/13 | 18 |
|  | NCT01968785 | Renal Denervation in Patients With Uncontrolled Blood Pressure | 15/08/13 | 20 |
|  | NCT02040155 | Real Time In-Vivo Dosimetry For Gynecologic Brachytherapy | 15/02/14 | 20 |
| **Nr.** | **ClinicalTrials.gov** | **Short Name** | **Trial  Start Date (D/M/Y)** | **Planned Accrual** |
|  | NCT02040493 | Intra-Operative Radiation Therapy Immediately Following Resection of Early Stage Breast Cancer | 15/09/08 | 75 |
|  | NCT02131805 | Electronic Skin Surface Brachytherapy for Cutaneous Basal Cell and Squamous Cell Carcinoma | 15/05/14 | 29 |
|  | NCT02271659 | Medical and Economic Evaluation for Intermediate-risk Prostate Cancer | 15/06/13 | 298 |
